# Nucleus accumbens projections: Validity and reliability of fiber reconstructions based on high‐resolution diffusion‐weighted MRI

**DOI:** 10.1002/hbm.25657

**Published:** 2021-09-16

**Authors:** Thilo Rusche, Jörn Kaufmann, Jürgen Voges

**Affiliations:** ^1^ Department of Stereotactic Neurosurgery Otto‐von‐Guericke University Magdeburg Magdeburg Germany; ^2^ Department of Radiology, Clinic of Radiology & Nuclear Medicine University Hospital Basel, University Basel Basel Switzerland; ^3^ Department of Neurology Otto‐von‐Guericke University Magdeburg Magdeburg Germany

**Keywords:** deep brain stimulation, diffusion‐weighted MRI, nucleus accumbens, projections

## Abstract

Clinical effects of deep brain stimulation are largely mediated by the activation of myelinated axons. Hence, increasing attention has been paid in the past on targeting white matter tracts in addition to gray matter. Aims of the present study were: (i) visualization of discrete afferences and efferences of the nucleus accumbens (NAc), supposed to be a major hub of neural networks relating to mental disorders, using probabilistic fiber tractography and a data driven approach, and (ii) validation of the applied methodology for standardized routine clinical applications. MR‐data from 11 healthy subjects and 7 measurement sessions each were acquired on a 3T MRI‐scanner. For probabilistic fiber tracking the NAc as a seed region and the medial prefrontal cortex (mPFC), anterior cingulate cortex (ACC), amygdala (AMY), hippocampus (HPC), dorsomedial thalamus (dmT) and ventral tegmental area (VTA) as target regions were segmented for each subject and both hemispheres. To quantitatively assess the reliability and stability of the reconstructions, we filtered and clustered the individual fiber‐tracts (NAc to target) for each session and subject and performed a point‐by‐point calculation of the maximum cluster distances for intra‐subject comparison. The connectivity patterns formed by the obtained fibers were in good concordance with published data from tracer and/or fiber‐dissection studies. Furthermore, the reliability assessment of the (NAc to target)‐fiber‐tracts yielded to high correlations between the obtained clustered‐tracts. Using DBS with directional lead technology, the workflow elaborated in this study may guide selective electrical stimulation of NAc projections.

## Graphical abstract

The nucleus accumbens (NAc), as a mesolimbic and dopaminergic relay station, and its efferent and afferent fiber‐projections play a central role within neuronal networks whose functions are impaired in various underlying neuro‐psychiatric disorders and could therefore be effective targets for the treatment of these disorders using deep brain stimulation (DBS). Therefore, the aim of the underlying work was a literature‐driven and diffusion‐weighted MRI‐based connectivity analysis, in vivo imaging, and subsequent inter‐ and intraproband comparison with respect to stability and reliability of the major neuronal fiber connections of the NAc. Overall, high anatomical validity, reliability, and stability were demonstrated for all considered and reconstructed NAc fiber connections, so that prospectively, from a clinical point of view, a valid preoperative fiber pathway reconstruction and thus a standardized preoperative intervention planning in the context of DBS would be possible and thus could improve the effectiveness and clinical outcome of this surgical method.

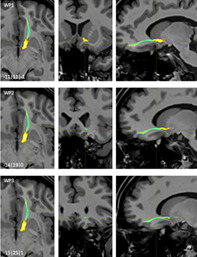

**Abbreviations**
A24: Area 24
ABmc: accessory basal nucleus mangnocellular division
ABp: accessory basal posterior
ABpc: accessory basal nucleus parvicellular division
ac: accumbofrontal fasciculus
ACC: anterior cingulate cortex
Ac: accumbofrontal fasciculus
Acr: anterior corona radiata
AD: anterior dorsal nucleus
aic: anterior limb of the internal capsule
AL: agranular insular
al: Ansa lenticularis
AMY: amygdala
APC: posterior limb of the anterior commissure
AV: anterior ventral nucleus
Ba: basal anterior
BLA: basolateral amygdala
BLM: basomedial amygdala
Bmc: basal nucleus magnocellular division
Bmec: basal nucleus mediocellular division
BNSM: bed nucleus of stria medullaris
BNST: bed nucleus of stria terminalis
Bp: basal posterior
Bpc: basal nucleus parvicellular division
CA: cornu ammonis
cc: corpus callosum
cC: cingulate cortex
Cd: nucleus caudatus
Cdm: medial caudate nucleus
Ce: central nucleus of amygdala
CG: pontomesencephalic central gray
CL: central lateral nucleus
ClN: central linear nucleus
CM: central nucleus of the meddling thalamus
COA: cortical nucleus amygdala
CPu: Claustrum‐Putamen
CS: superior central nucleus
CSD: constrained spherical deconvolution
CTA: cortical amygdaloid transition area
CVLM: caudal ventrolateral medulla
DB: diagonal band
DBS: deep brain stimulation
DH: dorsal hypothalamic area
dmT: dorsomedial thalamus
dmTN: dorsal midline thalamic nucleus
DTI: diffusion tensor imaging
DWI: diffusion‐weighted MRI
EN: endopiriform nucleus
EpN: endopeduncular nucleus
FLAIR: fluid attenuated inversion recovery
FOD: fiber orientation distribution
FuG: fusiform gyrus
GP: globus pallidus
HFec: hippocampal formation/entorhinal cortex
HFpc: hippocampal formation/perirhinal cortex
HFps: hippocampal formation/prosubiculum
HFS: hippocampal formation/subiculum
HPC: hippocampus
IAM: interanteromedial nucleus
IAPC: posterior limb of the anterior commissure
ICA: infralimbic cortical area
ifo: inferior longitudinal fasciculus
IF: interfascicular nucleus
IMD: intermediodorsal nucleus
IoC: islands of Calleja
LC: caudal linear nucleus
LC: locus coeruleus
lcdL: large cell devision lateral nucleus of amygdala
Ldl: dorsolateral division
LDT: laterodorsal tegmental nucleus
lenf: fasciculus lentiformis
LH: lateraler hypothalamus
LHA: lateral hypothalamic area
Lhb: lateral habenula
LP: lateral pulvinar nucleus
LP: lateroposterior nucleus
LR: rostral linear nucleus
LSN: lateral septal nucleus
Lvl: ventrolateral division
MDD: major depression disorder
mdTN: mediodorsal thalamic nucleus
MeAD: medial nucleus anterodorsal part
MePD: medial nucleus posterodorsal part
MFB: medial forebrain bundle
MI: primary motor cortex
MO(G): medial orbital cortex
MOPFC: medial orbital prefrontal cortex
mPFC: medial prefrontal cortex
MT: mesencephalic tegmentum
Na: nucleus arcuatus
NAc: nucleus accumbens
Nb: nucleus basalis
NTS: region of the dorsomedial medulla
NTS: nucleus tractus solitarius
NTS‐X: solitary‐vagal nuclear complex
OCA: cortical area
OCD: obsessive–compulsive disorder, orbital
OFC: orbital frontal cortex
OT: olfactory tubercle
Pa: paraventricular nucleus
PA: präoptic area
PB: parabrachial nucleus
PbN: parabigeminal nucleus
PC: perirhinal cortex
Pc: posterior commissure
pcN: paracentral nucleus
Pf: parafascicular nucleus of the thalamus
PFA: perifornical area
PFC: präfrontaler cortex
PH: posterior hypothalamic area
PiC: piriformer cortex
PN: paranigral nucleus
PR: peribrachial region
PT: parataenial nucleus
PTN: pedunculopontine tegmental nucleus
Pu: putamen
pvTN: paraventricular thalamic nucleus
racc: rostral anterior cingulate cortex
RAIC: rostral agranular insular cortex
Re: nucleus reuniens
RF: retrorubal field
RH: rhomboid nucleus
RN: raphe nucleus
Rs: rhinal sulcus
RTN: reticular thalamic nucleus
RVLM: rostral ventrolateral medulla
Sc: suprachiasmatic nucleus
scdL: small cell division lateral nucleus of amygdala
SI: substantia innominata
SFG: superior frontal gyrus
SG: gyrus rectus
sm: stria medullaris of thalamus
SN: substantia nigra
SSTI: substriatal terminal island
STN: nucleus subthalamicus
Str: stria terminalis
SuMn: supramamillary nucleus
sumx: supramammillar commissure
TM: tuberomamillary nucleus
TP: timepoint
TPP: nucleus tegmenti pedunculopontinus
unc: fasciculus uncinatus
VC: ventral capsule
VDB: ventral branch of the diagonal band
vHC: ventral hippocampus
VMH: ventromedial hypothalamic nucleus; dorsomedial part, ventrolateral part
VO: ventral orbital cortex
VOI: volume of interest
VP: ventrales pallidum
VT/VS: ventral striatum
VTA: ventral tegmental area
WP: waypoint

## 1 INTRODUCTION

The NAc as the major component of the ventral striatum (VS) is a key relay in the mesolimbic, dopaminergic reward system with strong connections to the amygdala (AMY), dorsomedial thalamus (dmT), ventral tegmental area (VTA), hippocampus (HPC) and to the neocortex comprising the anterior cingulate cortex (ACC) and the orbital and the medial prefrontal cortex (OFC, mPFC) (Haber & McFarland, 1999).

Brain areas such as the dorsal ACC (dACC), OFC, ventro‐medial (vm) PFC and AMY were specifically related to the pathophysiology of psychiatric disorders (Haber & Behrens, 2014; Ressler & Mayberg, 2007). Hence NAc and VS are supposed to be a major hub within these networks and have already been used as a target to treat obsessive compulsive disorder (OCD), major depressive disorder (MDD) or addiction with deep brain stimulation (DBS) (Bewernick, Kayser, Sturm, & Schlaepfer, 2012; Denys et al., 2010; Sturm et al., 2003; Voges, Muller, Bogerts, Munte, & Heinze, 2013). In addition, NAc‐DBS improved substantially partial epilepsy in an open labeled case series and a randomized controlled case series (Kowski et al., 2015; Schmitt et al., 2014).

The clinical efficacy of DBS bases mainly upon the activation of myelinated axons leading to local but also network‐wide electrical and neurochemical effects with modification of the oscillatory activity of neurons, synaptic plasticity or the degree of synchronization between different brain areas (Herrington, Cheng, & Eskandar, 2016; Udupa & Chen, 2015). Hence, the first goal of the present study was to visualize in the brain of healthy individuals selectively those neural projections connecting the NAc with the abovementioned brain regions using diffusion‐weighted MR imaging (DWI) and probabilistic fiber tractography. The target regions were selected on the basis of a detailed data analysis from anatomical tracer studies (brain of rodents, pigs or non‐human primates) and from fiber‐dissection studies (human brains) with regard to the functionally and anatomically most important NAc afferents and efferents. The second goal of the present study was to demonstrate the validity and reliability of our methodological approach and to develop a standardized workflow for the routine application in patients.

## 2 MATERIALS AND METHODS

### 2.1 Literature review

The Pubmed‐based literature research was performed using the following search combinations (abstract/title) up to and including June 2021: “NAc AND connections,” “NAc AND efferents,” “NAc AND afferents,” “NAc AND DTI,” “NAc AND tract tracing,” “NAc AND projections” and “NAc AND networks.” The search was limited to publications in English language and anatomical studies performed on non‐human primates, pigs, mice, or rats using either chemical fiber tract‐tracing or autoradiography. We tabularly summarized the collected data and compared them with the database on www.bams1.org. Afterwards a graphic illustration was implemented with the freeware tool *RAWGraphs* on www.rawgraphs.io. To define clinically relevant NAc‐connections we finally identified those projection areas, whose dysfunction is supposed to be causative related to the symptoms of OCD, MDD or addiction (Giacobbe, Mayberg, & Lozano, 2009; Heimer, 2003; Milad & Rauch, 2012).

### 2.2 MRI‐datasets and segmentation

#### 2.2.1 MRI data acquisition

**FIGURE 1:**
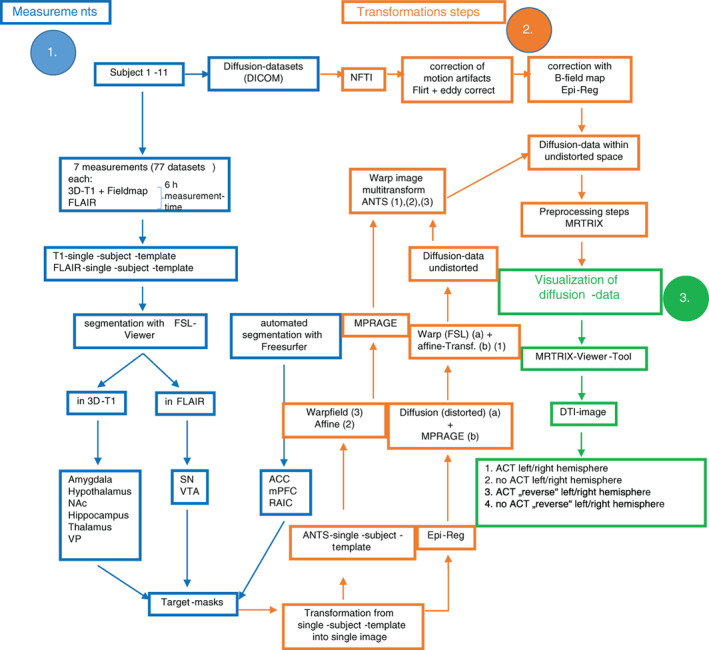
Preprocessing and analysis pipeline

Eleven healthy subjects (five female, six male, ten right‐handed, one left‐handed, average age: 29.1 years) gave their informed consent (positive vote from the ethics committee of the Otto‐von‐Guericke‐University Magdeburg; approval number: 106/98) for cranial MRI‐examinations. In each case, we repeated the examinations seven times as separate sessions using a Siemens *MAGNETOM Prisma* 3T‐scanner, the software *Syngo D13D* and a standard 64‐channel phased array imaging coil in receive mode (for specific measurement parameters see Table 1; for more detailed explanation of performed steps see Figure 1). To increase inter‐subject reproducibility in position and minimize motion a pillow was placed surrounding the sides and the back of the head. The field of view was aligned in all diffusion scans to the anterior commissure (AC)—posterior commissure (Pc) (AC‐PC)‐line. Within each session, a 3D‐T1‐weighted volume (MPRAGE), a FLAIR‐weighted image, a sequence of diffusion‐weighted volumes, and a GRE field map of the volume were acquired. The diffusion‐weighted volumes were acquired along 60 noncollinear diffusion directions. We allowed for parallel acquisition of independently reconstructed images using generalized auto calibrating, partially parallel acquisitions or *GRAPPA* (Griswold et al., 2002), with acceleration factor of 3 and 57 reference lines. Each diffusion direction was scanned twice (direction vector and inverted direction vector). Ten diffusion‐weighted volumes followed an unweighted volume. In total, 120 diffusion‐weighted volumes (*b* = 1,000 s/mm^2^) and 13 non‐diffusion‐weighted volumes (*b* = 0 s/mm^2^) were obtained in each session. The overall measurement time for each individual was about 6 hr.

**TABLE 1:** Measuring parameters and used MRI‐sequences

	3D‐T1	dMRI	B0‐mapping	FLAIR
Sequence type	MPRAGE	SE‐EPI	GRE	spcir
FOV (field of view) in mm	256 × 256	220 × 220	220 × 220	230 × 230
Resolution in mm	0.8 × 0.8 × 0.8	1.6 × 1.6 × 1.6	1.6 × 1.6 × 2.0	0.9 × 0.9 × 0.9
TR (repetition time) in ms	2,600	10,200	720	5,000
TE (echo time) in ms	4,50	49	4,92	167
TI (inversion time) in ms	1,100			1,800
Flip‐angle in degree	7		60	
Bandwidth in Hz/pixel	140	2,012	739	501
Time of acquisition	13 min:52 s	23 min:38 s	3 min:20 s	23 min:50 s

#### 2.2.2 Region‐of‐interest (target) segmentation

We defined clinically relevant targets as mentioned above and segmented these targets. NAc, HPC, AMY and dmT were manually segmented within the T1‐weighted single‐subject‐template, the VTA within the FLAIR‐single‐subject‐template separately for the left and right hemisphere using *FSL‐Viewer* (FMRIB Software Library, Release 5.0.9 [Jenkinson, Beckmann, Behrens, Woolrich, & Smith, 2012]; The University of Oxford). Reference for the definition of landmarks for each target was the “Atlas of the human brain” (Mai, Voss, & Paxinos, 2008). A computer‐based, automated target segmentation was carried out for the cortical areas ACC and mPFC using a *Freesurfer* software package (version 5.3) (Dale, Fischl, & Sereno, 1999).

### 2.3 Diffusion‐weighted MRI based fiber‐reconstructions and visualization of selective NAc‐connections

#### 2.3.1 MRI‐data preprocessing and target‐mask transformation

*DICOM* to *NIFTI* conversion was carried out using *MRIcron's dcm2nii* (http://people.cas.sc.edu/rorden/mricron). Next, diffusion data were preprocessed using *dwidenoise* and *mrdegibbs* with a program package of *MRtrix* ([Kellner, Dhital, Kiselev, & Reisert, 2016; Tournier et al., 2019]; www.mrtrix.org). To correct for eddy‐current‐induced distortions, the diffusion‐weighted images were registered to a corresponding b0‐image based on a 12‐dof affine transformation using FSL‐tool *eddy_correct* with spline interpolation (Graham, Drobnjak, & Zhang, 2016). To account for head movement, an affine transformation from each block's non‐diffusion‐weighted volume to the first b0‐image using FSL‐tool *flirt* (Jones & Cercignani, 2010; Smith et al., 2004). The DWI data of each block were then realigned based on these transformations. Geometric distortions induced by magnetic field inhomogeneities were corrected based on the GRE field map, and the diffusion data were registered to the corresponding T1‐weighted image. These steps (EPI distortion correction and EPI‐to‐MPRAGE registration) were performed simultaneously using FSL‐tool *epi_reg*. Finally, a mask consisting of non‐weighted diffusion‐data was calculated only containing the brain (brain‐mask) with FSL‐tool *bet*. In the following steps based on the MRtrix3 software package using *dwi2response* response functions from the preprocessed diffusion‐weighted images and fiber orientation distributions (FOD) with *dwi2fod* using constrained spherical deconvolution (CSD) were estimated.

The target‐masks were transformed from each T1/FLAIR‐single‐subject‐template volume into the corresponding subject diffusion‐weighted data space with the program package *ANTs* (WarpImageMultiTransform [Avants et al., 2010]) as a composite function of three (T1‐template ➔ MPRAGE, MPRAGE ➔ diffusion‐weighted data [affine + nonlinear transformation]) (T1) respective four transformation steps (FLAIR) (FLAIR ➔ T1‐template transformation additionally).

#### 2.3.2 Fiber‐tracking: Graphic visualization of DWI‐datasets with MRtrix

All generated target masks could be used as a fiber‐tracking seed or target region. Graphical notation of the datasets was realized with the program *MRtrix*. Using *tckgen*, a minimum of 100,000 and a maximum of 10^7^ starts were generated outgoing from the start regions. A probabilistic, unidirectional fiber tracking procedure and standard parameters were used (algorithm *iFOD2*, Proc. Intl. Soc. Mag. Reson. Med. 18 [2010]). Moreover, versions with (ACT‐version (information of the distribution of white and gray matter) (Smith, Tournier, Calamante, & Connelly, 2012)) or without anatomic information were considered. For every seed to target connection, we defined a maximum fiber‐length: ACC 55 mm, AMY 40 mm, HPC 65 mm, mPFC 55 mm, dmT 30 mm and VTA 30 mm.

Next, we generated FA‐single‐subject‐templates using the FA‐maps of timepoint 1–7 for each subject (*ANTs, Multivariate TemplateConstruction 2*). Then, the fiber‐coordinates were transformed to the single‐subject‐space (*ANTs* and *MRtrix* with command *tcktransform*) utilizing the linear (affine) and nonlinear (Warp‐fields) transformations.

Afterwards, paths with similar properties (i.e., trajectories, length) were grouped into clusters of paths using a clustering algorithm implemented in Matlab (Mathworks, Natick, MA) with the following criteria:

- The start‐ and endpoint as well as two equidistant points along the path must not exceed a spatial Euclidian distance of 10 mm.
- The difference in path‐lengths must not be greater than 10%.

With some NAc‐target connections (mPFC, ACC and HPC) a so‐called filtering of the fiber‐tracts was necessary before the clustering process. This was always the case when fiber‐tracts (even after optimizing the maximum fiber‐length) had partially inhomogeneous path courses over contralateral structures (especially anterior commissure) or several path segments and the clustered fiber‐tracts would therefore be inconsistent or significantly divergent. In detail (like the ROI‐definition for the target masks), a fiber‐tract‐specific ROI for both hemispheres was segmented into the FA‐single‐subject‐templates. The ROIs (2 × 2 pixels) were segmented in coronal slices for both hemispheres:

1. Within the fiber‐pathway of the anterior corona radiata (acr) shortly before the distribution in the gyrus rectus (SG) and superior frontal gyrus (SFG) for the NAc‐mPFC‐connection.
2. At the level of the crossing point of the ventral/dorsal ACC at the level of the genu corporis callosi for the NAc‐ACC‐connection.
3. Within the distal part of the stria terminalis (str) for the NAc‐HPC‐connection.

The visualization of the paths was conducted with the MRtrix viewer *mrview* using seven single measurements/datasets of a representative sample subject (see Figures 3, 4, 5, 6, 7, 8, 9, 10). In order to generate comparable coordinates with regard to the common literature (especially in comparison to the Mai‐Atlas [Mai et al., 2008]), the T1‐template was transferred to a coordinate system whose *y*‐axis corresponds to the AC‐PC‐line with the AC as origin (0|0|0) (*x*‐axis form left to right and *z*‐axis from inferior to superior). All defined waypoints (WP; representative and path‐specific point of fiber‐tract) (see Figures 3, 4, 5, 6, 7, 8, 9, 10) are denoted in this system. In particular, the distance between the AC and PC of the brain of the Mai‐Atlas (approx. 28 mm) was comparable with that of the sample subject (approx. 26 mm). This additionally ensures good transferability of the data to the generally valid Mai‐Atlas.

### 2.4 Evaluation of fiber‐tracts reliability and stability

The statistical evaluation based on the 924 NAc to target single‐fiber‐connections of subject 1–11 for timepoint 1–7 and both hemispheres with the ACT‐algorithm (see above).

#### 2.4.1 Intra‐subject‐comparison

Optimal stereotactic treatment planning depends to a large proportion on the quality of the underlying MRI‐images, in the present case on the reliability and stability of the calculated fiber‐tracts. To determine this variable, we performed a quantitative comparison of the single‐fiber‐connections for all NAc to target pathways and a qualitative‐visual comparison exemplarily for the NAc to mPFC connections.

For the qualitative‐visual‐comparison, we visualized the NAc to mPFC fiber‐connections for each subject (1–11) and timepoint (1–7) in the left hemisphere using *MRview*. Next, we determined the AC on axial slices of the generated MRI‐datasets as an anatomical reference point. Finally, with the help of *Inkscape* (version 0.92.2) screenshots of the displayed fiber‐connections were tabularly arranged (row: subject 1–11; column: timepoint 1–7).

Quantitative comparison was performed by generating a trajectory for each clustered main‐pathway (pathway with the most identic fiber‐connections) (see Section 2.3.2) of each NAc‐target connection (mPFC, ACC, HPC, AMY, VTA and dmT) for each session 1–7 and each subject 1–11. The trajectory was calculated by determining the centroid‐path from the coordinates of all individual paths within the cluster. In a next step the shortest trajectory was selected and the Euclidian distances to each other trajectories were calculated point‐by‐point. The different distances were then saved and plotted (*x*‐axis: steps in the clustered paths [0.8 mm per step]); *y*‐axis: distance to the shortest cluster in mm). Finally, the following values were also calculated and summarized in a table (see Section 3.2.1 and Figure 15):

1. The maximum Euclidian distances of single‐clusters to the shortest single‐cluster (maximum distance of a single‐cluster to the shortest single‐cluster per session [measurement timepoint] and subject and NAc‐target connection per hemisphere).
2. The average maximum Euclidian distances of single‐clusters to the shortest single‐cluster (average maximum distances of single‐clusters for session 1–7 of a subject 1–11 per NAc‐target connection and hemisphere).
3. Maximum mean distances (average maximum distance of single‐clusters for all subjects 1–11 and sessions 1–7 per NAc‐target connection and hemisphere).

#### 2.4.2 Inter‐subject‐comparison using the example of the NAc to mPFC fiber‐connections

Accuracy and fidelity to reality of the reconstruction of NAc fiber‐connections depends on the identification and accordance of fiber‐tract‐specific waypoints. To determine these variables, we performed a qualitative‐visual inter‐subject‐comparison of the NAc to mPFC single‐fiber‐connections of subject 1–11 by preparing and analyzing a tabularly overall image as described in Section 2.3.1.

## 3 RESULTS

### 3.1 Literature analysis

**FIGURE 2:**
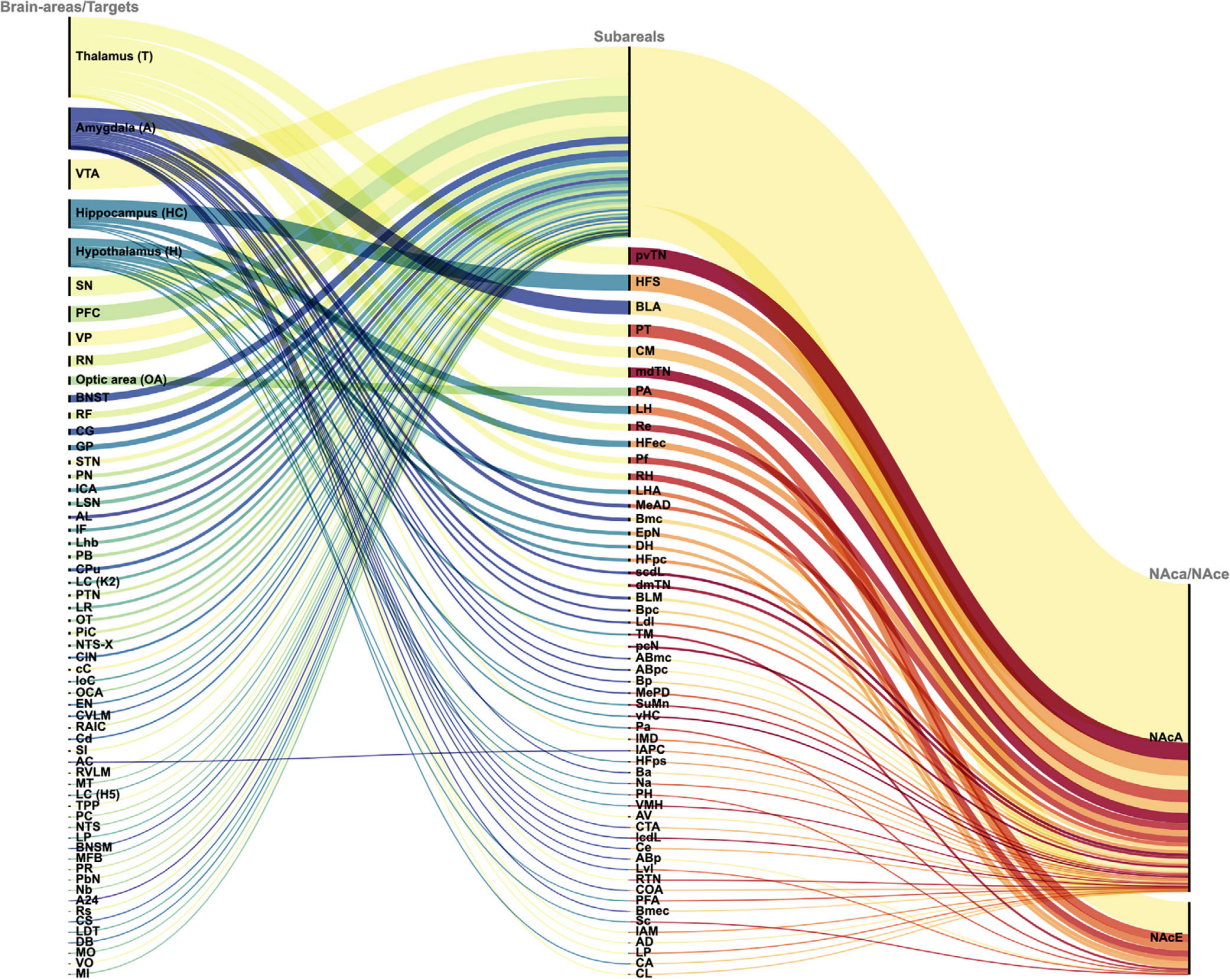
Quantitative visualization of NAc‐projections according to the results of published tracer studies

The defined search combinations leaded to the following hits: “NAc AND connections”: 397, “NAc AND efferents: 113, “NAc AND afferents”: 379, “NAc AND DTI”: 53, “NAc AND tract tracing”: 48, “NAc AND projections”: 979, and “NAc AND networks”: 401. After filtering 207 manuscripts remained. 58 out of search combination one, 6 out of two, 47 out of three, 0 out of four, 12 out of five, 82 out of six and 1 out of seven. From each of the remained publications we extracted the observed NAc projections and documented them tabularly. In a second step, we displayed the numbers of the various NAc‐afferents (NAca) and efferents (NAce) in a graph (Figure 2). The width of the individual lines, graphically represented in different colors, was greater the more often the corresponding single‐connection was described in the manuscripts we examined. Thalamus, Amygdala, Hypothalamus, Hippocampus, Optic area (OA) and anterior Commissure (AC) were additionally divided into sub‐regions. The most frequently described connections were from NAc to Thalamus (151), AMY (79), VTA (56), HPC (54), Hypothalamus (54), SN (36), PFC (30), and VP (26).

### 3.2 Diffusion‐weighted‐MRI based fiber‐reconstructions and visualization of selective NAc‐connections

We were able to construct a fiber‐tract with *MRtrix* including fiber‐tracking with or without ACT as well as reverse tracking (target as seed region, NAc as target region) for each individual NAc‐to‐target‐connection. In total, we generated 3,080 fiber‐tracts. Independent from the applied method (with ACT, without ACT and reverse) each NAc‐to‐target connection lead to qualitatively the same fiber‐patterns which were thoroughly consistent with the published anatomic data. The following description of fiber‐pathways connecting the NAc with the AMY, ACC, dmT, HPC, mPFC and VTA summarizes the information, which we extracted from MRI‐based fiber‐tracking. For the description of the 3D‐orientation we used the forebrain axis (Forel's axis) with the directional terms rostral‐caudal/posterior and dorsal‐ventral, respectively (Forel et al., 1875). The indicated anatomical landmarks refer to the “Atlas of the human brain” published by Mai & Paxinos (Mai et al., 2008).

#### 3.2.1 NAc to AMY (Figure 3)

**FIGURE 3:**
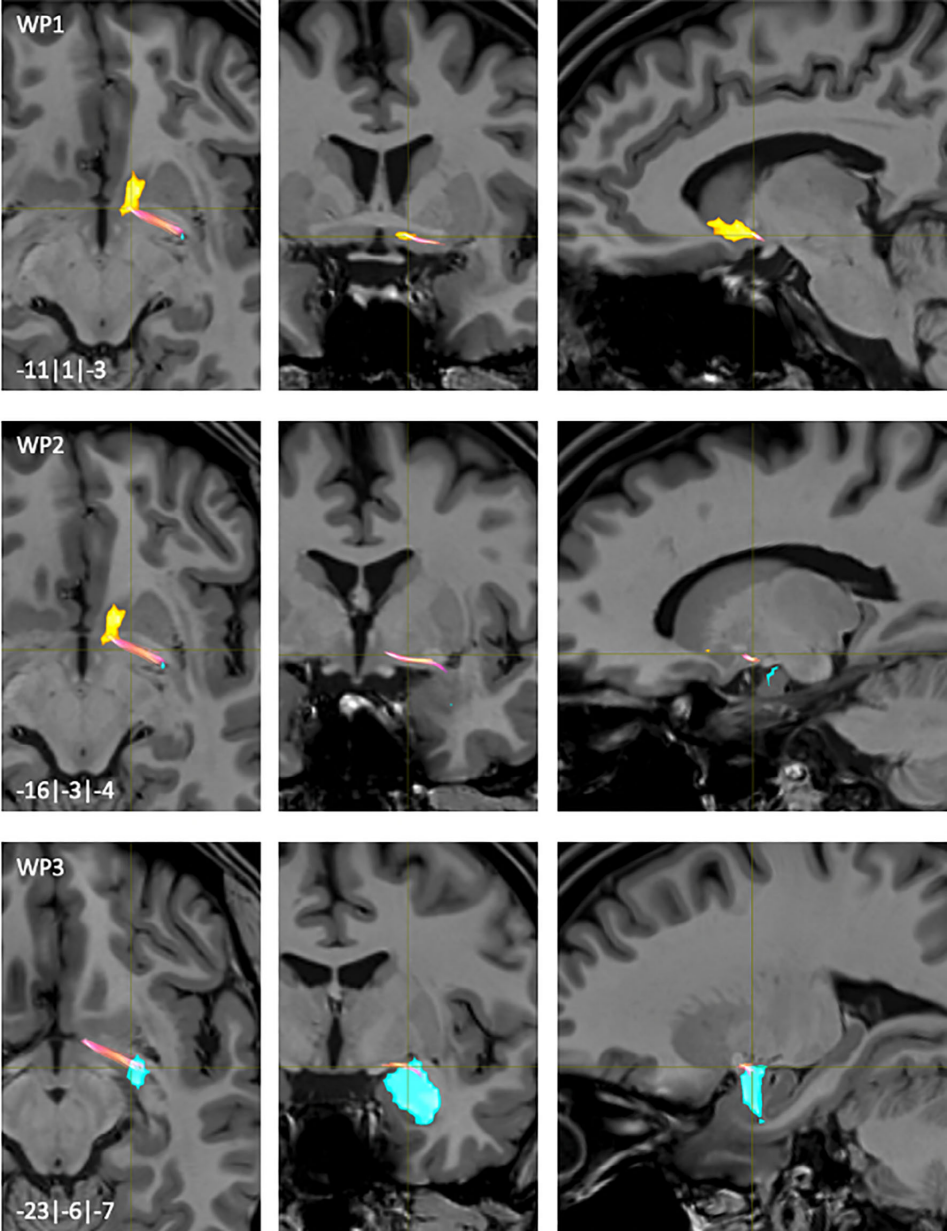
NAc to Amygdala fiber‐tract for left hemisphere: Waypoints 1–3 and distance to AC (0|0|0) in mm (*x*|*y*|*z*) = (left(−)/right|posterior(−)/anterior|inferior(−)/superior); yellow surface: NAc, light blue surface: Amygdala

The main fiber bundles originated directly from posterior parts of the NAc (WP1, Figure 3). Smaller fibers originating from central parts of the NAc joined with these main bundles throughout their further common course. Afterwards, the fibers passed posterolateral into the Ansa lenticularis‐pathway (WP2; Figure 3). Most of the fibers entered posterolateral parts of the AMY projecting onto the basal and central amygdala nuclei (WP3, Figure 3).

#### 3.2.2 NAc to ACC (Figure 4)

**FIGURE 4:**
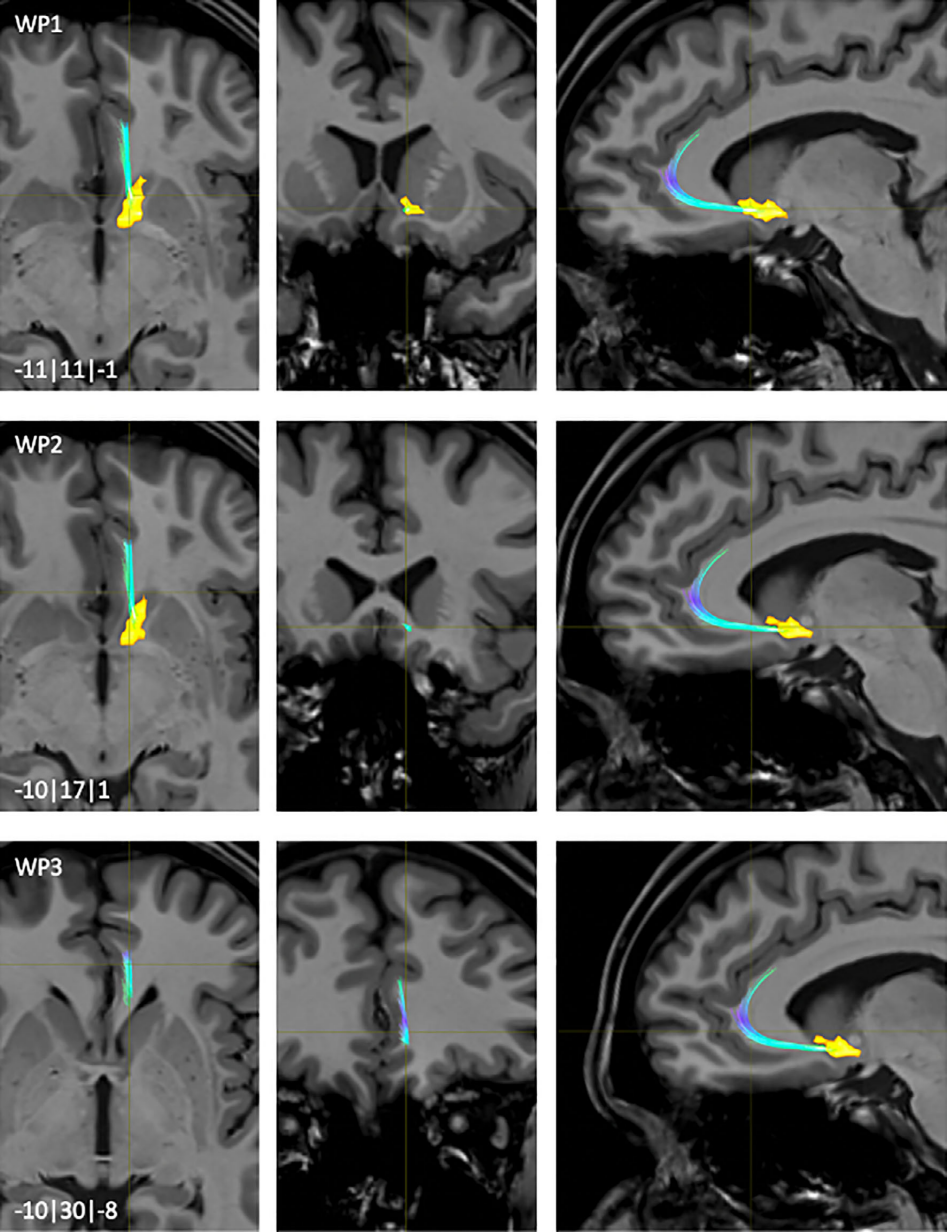
NAc to ACC fiber‐tract for left hemisphere: Waypoints 1–3 and distance to AC (0|0|0) in mm (*x*|*y*|*z*) = (left(−)/right|posterior(−)/anterior|inferior(−)/superior); yellow surface: NAc

The majority of fibers connecting the NAc with the ACC originated in rostro‐ventro‐medial parts of the NAc (WP1, Figure 4). Then, the fibers followed the ventral branch of the diagonal band (VDB) to the rostral fiber‐parts of the Corpus callosum (cc) (WP2, Figure 4). From the rostrum of the cc the fibers reached the Gyrus cinguli performing the typically falciform configuration of that gyrus (WP3, Figure 4).

#### 3.2.3 NAc to mdT (Figure 5)

**FIGURE 5:**
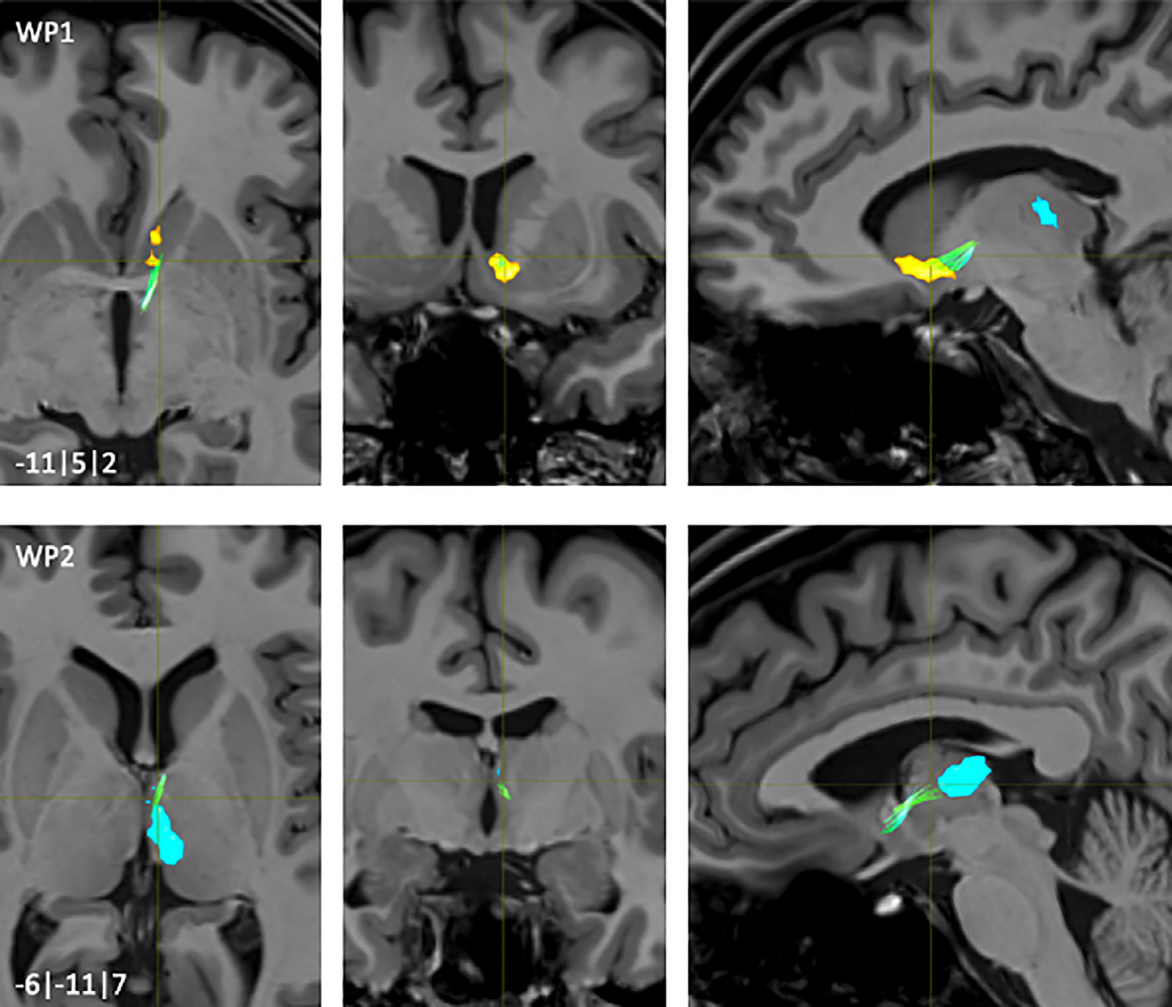
NAc to mdT fiber‐tract for left hemisphere: Waypoints 1–2 and distance to AC (0|0|0) in mm (*x*|*y*|*z*) = (left(−)/right|posterior(−)/anterior|inferior(−)/superior); yellow surface: NAc; light blue surface: dmT

We identified a fiber‐pathway, which originated in rostrolateral parts of the NAc and had a straight course posterior and parallel to the anterior limb of internal capsule (aic) (WP1, Figure 5). The fibers of this pathway continued inside the lateral wall of the third ventricle in dorsal‐posterior direction projecting mainly onto the paraventricular thalamic nucleus (pvTN) and stria medullaris of thalamus (sm). This pathway entered the dorsal and ventral parts of the medial thalamus from medial (WP2, Figure 5).

#### 3.2.4 NAc to HPC (Figures 6 and 7)

**FIGURE 6:**
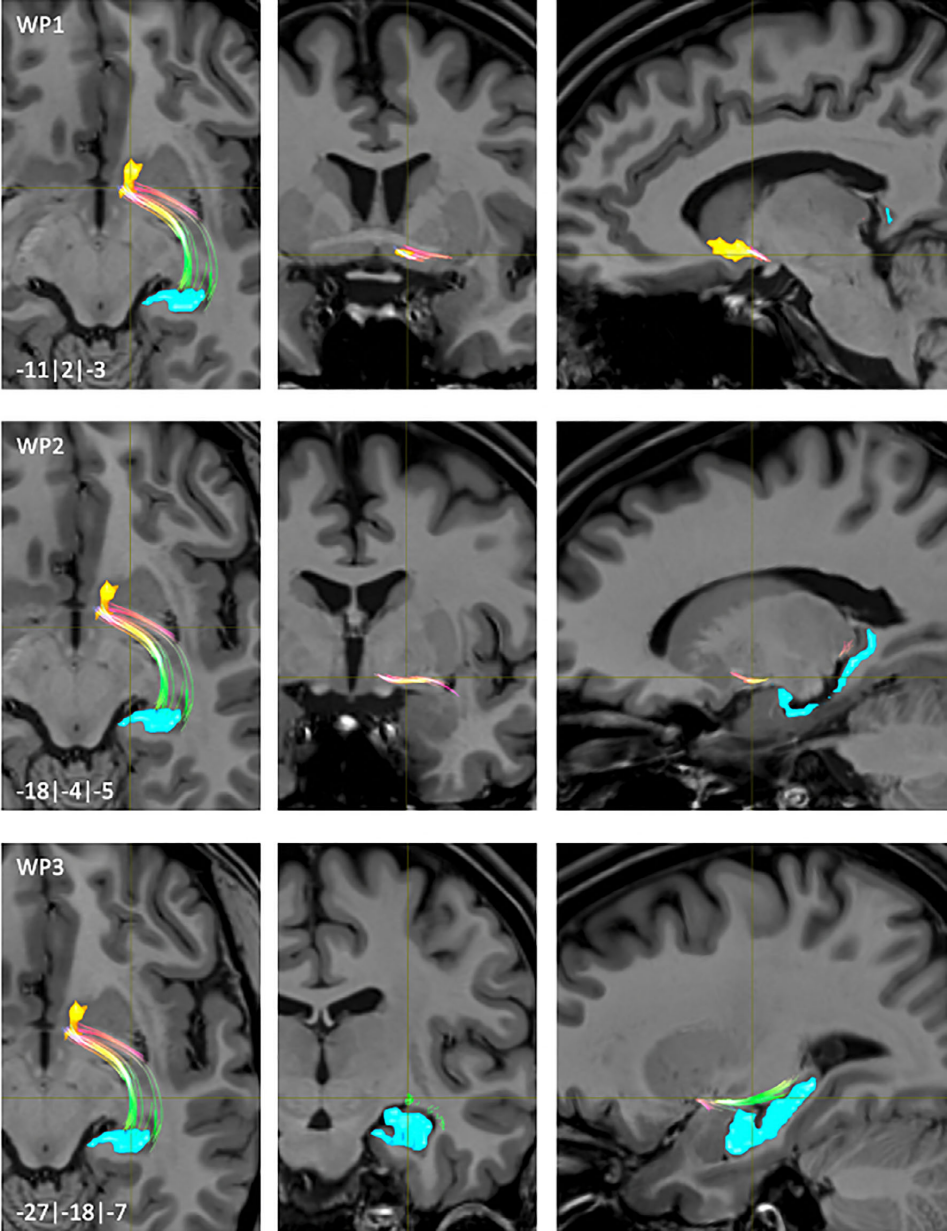
NAc to hippocampus fiber‐tract for left hemisphere: Waypoints: 1–3 and distance to AC (0|0|0) in mm (*x*|*y*|*z*) = (left(−)/right|posterior(−)/anterior|inferior(−)/superior); yellow surface: NAc, light blue surface: hippocampus

**FIGURE 7:**
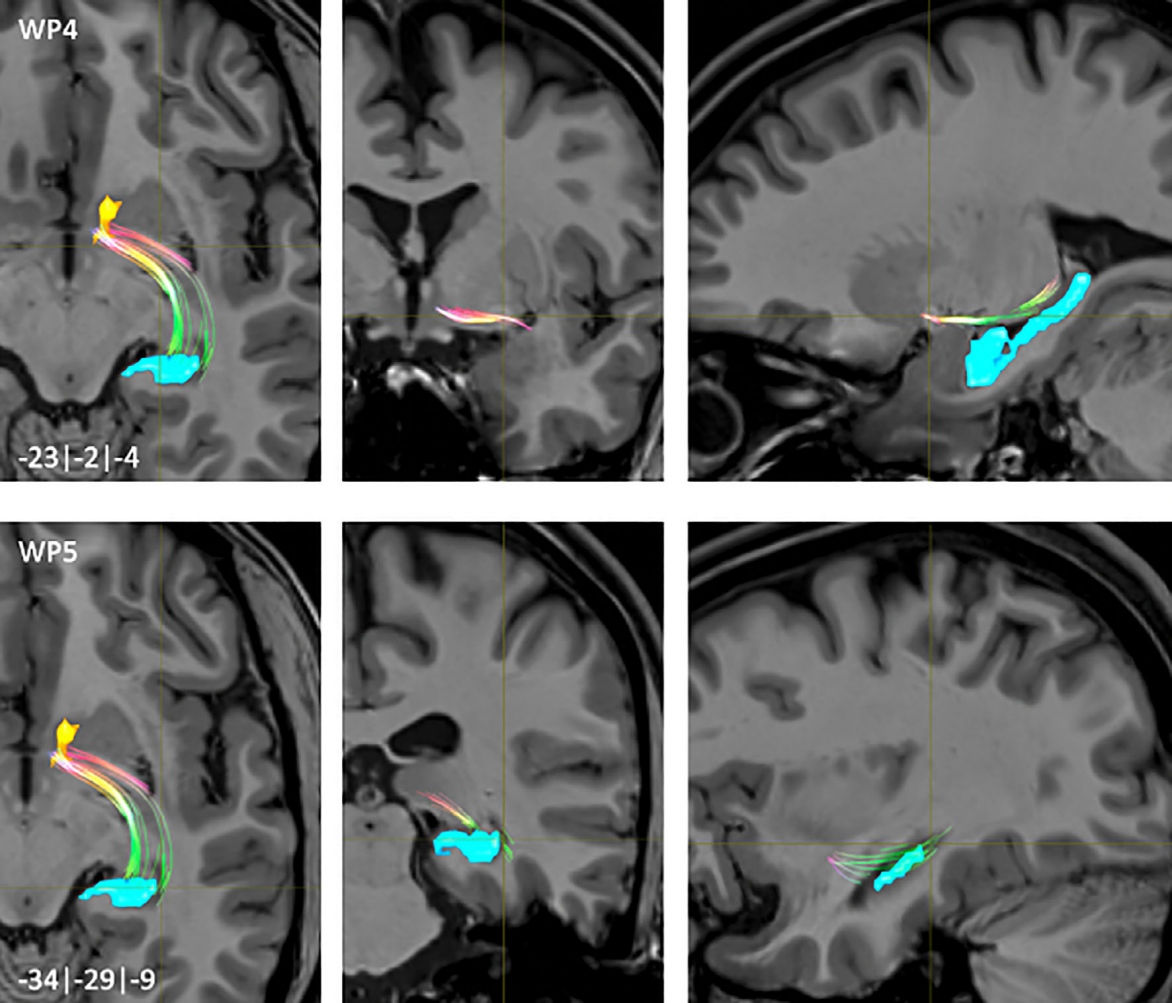
NAc to hippocampus fiber‐tract for left hemisphere: Waypoints: 4–5 and distance to AC (0|0|0) in mm (*x*|*y*|*z*) = (left(−)/right|posterior(−)/anterior|inferior(−)/superior); yellow surface: NAc, light blue surface: hippocampus

The NAc to HPC fibers emerged from dorsocaudal areas of the NAc (WP1, Figure 6) with a short lateral course together with the AC. From that point on they were divided in two tracts. The medial one went caudomedially passing the Fasciculus lenticularis (lenf) (WP2, Figure 6) and followed fibers of the Stria terminalis (str) (WP3, Figure 6). Another more lateral fiber‐tract passed from the AC ventro‐lateral within the fiber‐pathway of the Ansa lenticularis (al) (WP4, Figure 7) before terminating mainly into posterolateral parts of the HPC (WP5, Figure 7).

#### 3.2.5 NAc to mPFC (Figures 8 and 9)

**FIGURE 8:**
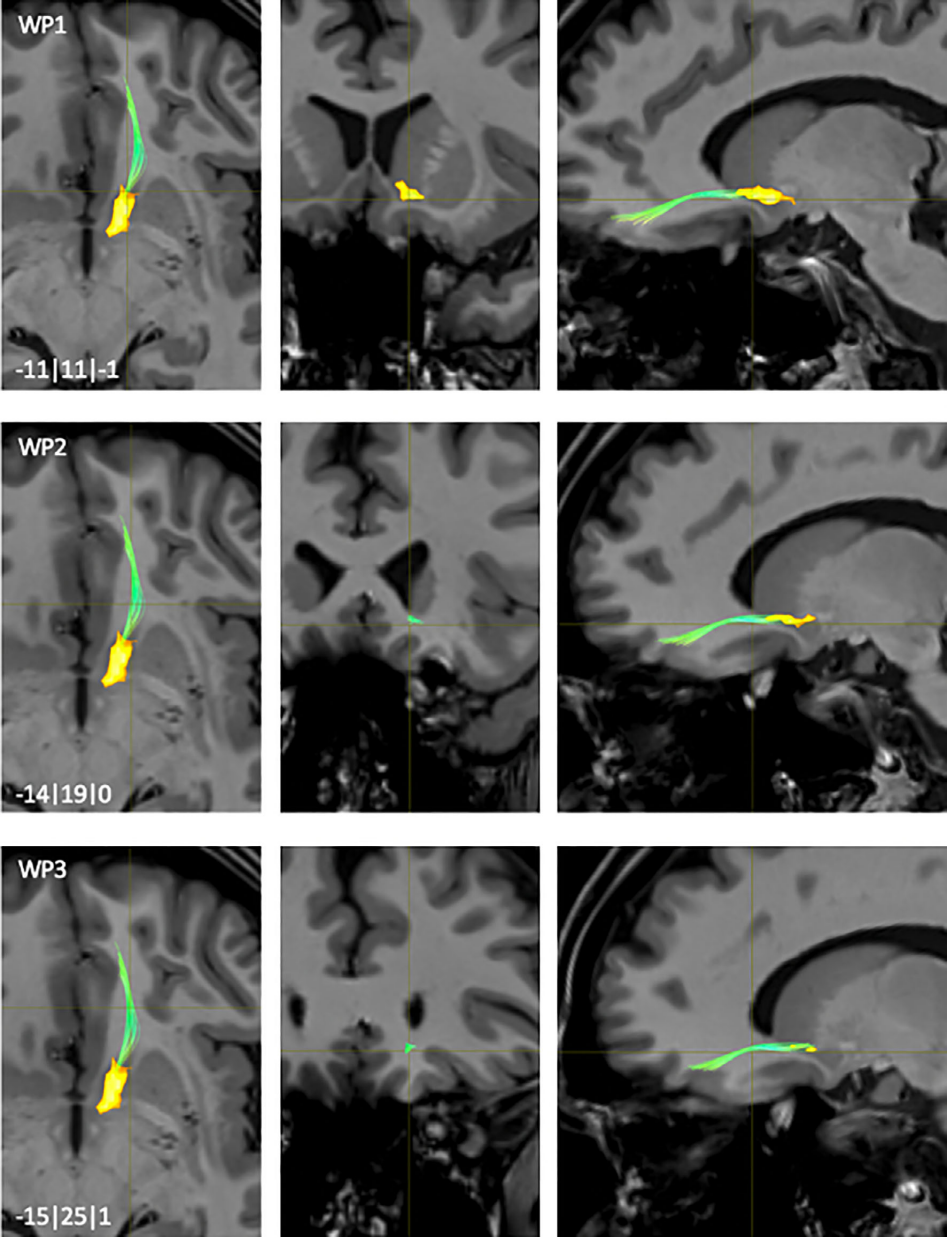
NAc to mPFC fiber‐tract for left hemisphere: Waypoint: 1–3 and distance to AC (0|0|0) in mm (*x*|*y*|*z*) = (left(−)/right|posterior(−)/anterior|inferior(−)/superior); yellow surface: NAc

**FIGURE 9:**
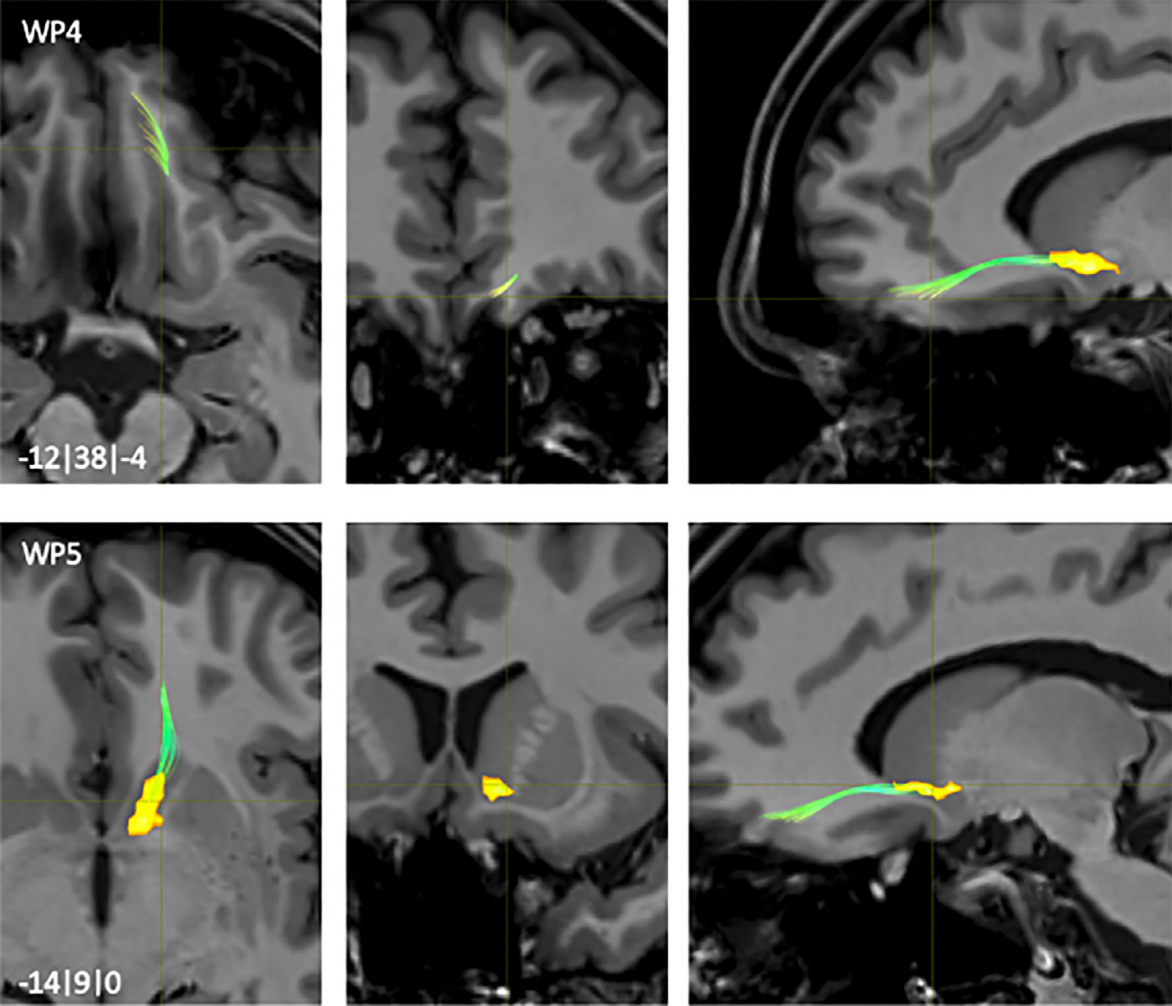
NAc to mPFC fiber‐tract for left hemisphere: Waypoint: 4–5 and distance to AC (0|0|0) in mm (*x*|*y*|*z*) = (left(−)/right|posterior(−)/anterior|inferior(−)/superior); yellow surface: NAc

The connections from the NAc to the mPFC were divided into a medial and a lateral fiber‐pathway (WP1, Figure 8). The medial pathway projected onto the accumbo‐frontal fascicle (ac) (Rigoard et al., 2011). Starting from ventromedial, predominantly from rostromedial parts of the NAc, the course of this tract was in rostral direction (WP2, Figure 8). The majority of the fibers approached the Gyrus rectus (SG) and superior frontal gyrus (SFG) via fibers of the anterior corona radiata (Acr) (WP3/4, Figures 8 and 9). Some fibers also ended within parts of the medial orbital gyrus (MOG).

The lateral fiber‐pathway started mainly from dorsolateral parts of the rostral half of the NAc (WP5, Figure 9). After their rostral course, the main part of the fibers approached the MOG in the region of the medial orbital PFC (MOPFC).

#### 3.2.6 NAc to VTA (Figure 10)

**FIGURE 10:**
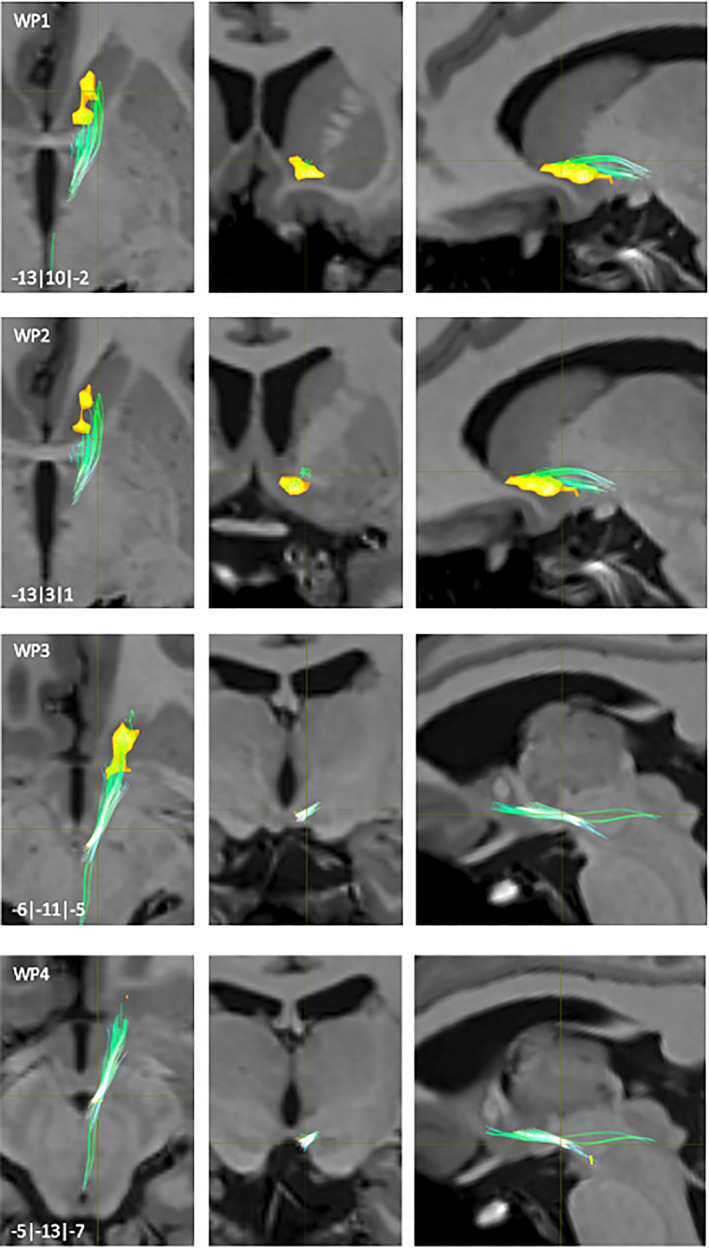
NAc to VTA fiber‐tract for left hemisphere: Waypoint: 1‐3 and distance to AC (0|0|0) in mm (x|y|z) = (left(−)/right | posterior(−)/anterior | inferior(−)/superior); yellow surfaces: NAc, VTA

These connections had starting points in dorso‐caudo‐lateral and posterior compartment of the NAc (WP1, Figure 10). Fibers emerging from the dorso‐caudo‐lateral NAc extended straightly into posterior direction within fibers of the ventral part of the anterior limb of the internal capsule (vaic) located between the NAc and ventral putamen (WP2, Figure 10) reaching more ventro‐posteriorly the dorsal hypothalamus (DH). Fibers emerging from the posterior NAc merged with the dorso‐rostro‐lateral fibers at the level of the DH (WP3, Figure 10) before ending together within the VTA. Another third small fiber‐pathway became visible on coronal slices emerging from the supramammillar commissure (sumx) to rostro‐dorso‐medial parts of the VTA (WP4, Figure 10). Some of these fibers projected onto the mesencephalon but did not end in the VTA. These structures represented most likely artifacts.

### 3.3 Fiber‐tract reliability and stability

#### 3.3.1 Intra‐subject‐comparison

In general, the qualitative‐visual intra‐subject‐comparison of the NAc to mPFC fiber‐connections and quantitative intra‐subject‐comparison of all NAc to target connections (NAc ➔ mPFC, NAc ➔ ACC, NAc ➔ HPC, NAc ➔ AMY, NAc ➔ VTA, NAc ➔ dmT) could be executed for each session (measurement timepoint) 1–7 and subject 1–11.

**FIGURE 11:**
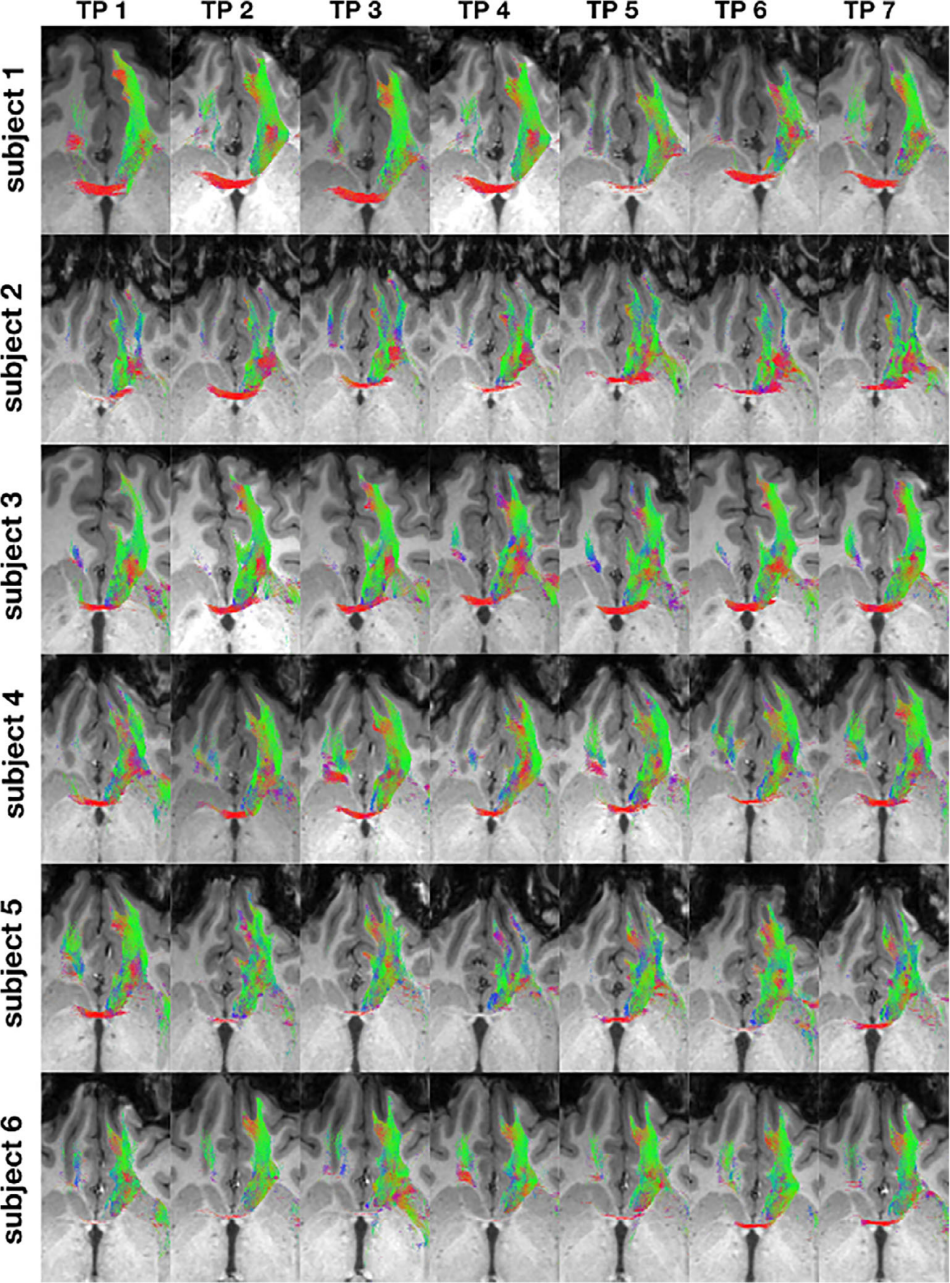
NAc to VTA fiber‐tract for left hemisphere: Waypoint: 4 and distance to AC (0|0|0) in mm (x|y|z) = (left(−)/right | posterior(−)/anterior | inferior(−)/superior); yellow surfaces: NAc, VTA

**FIGURE 12:**
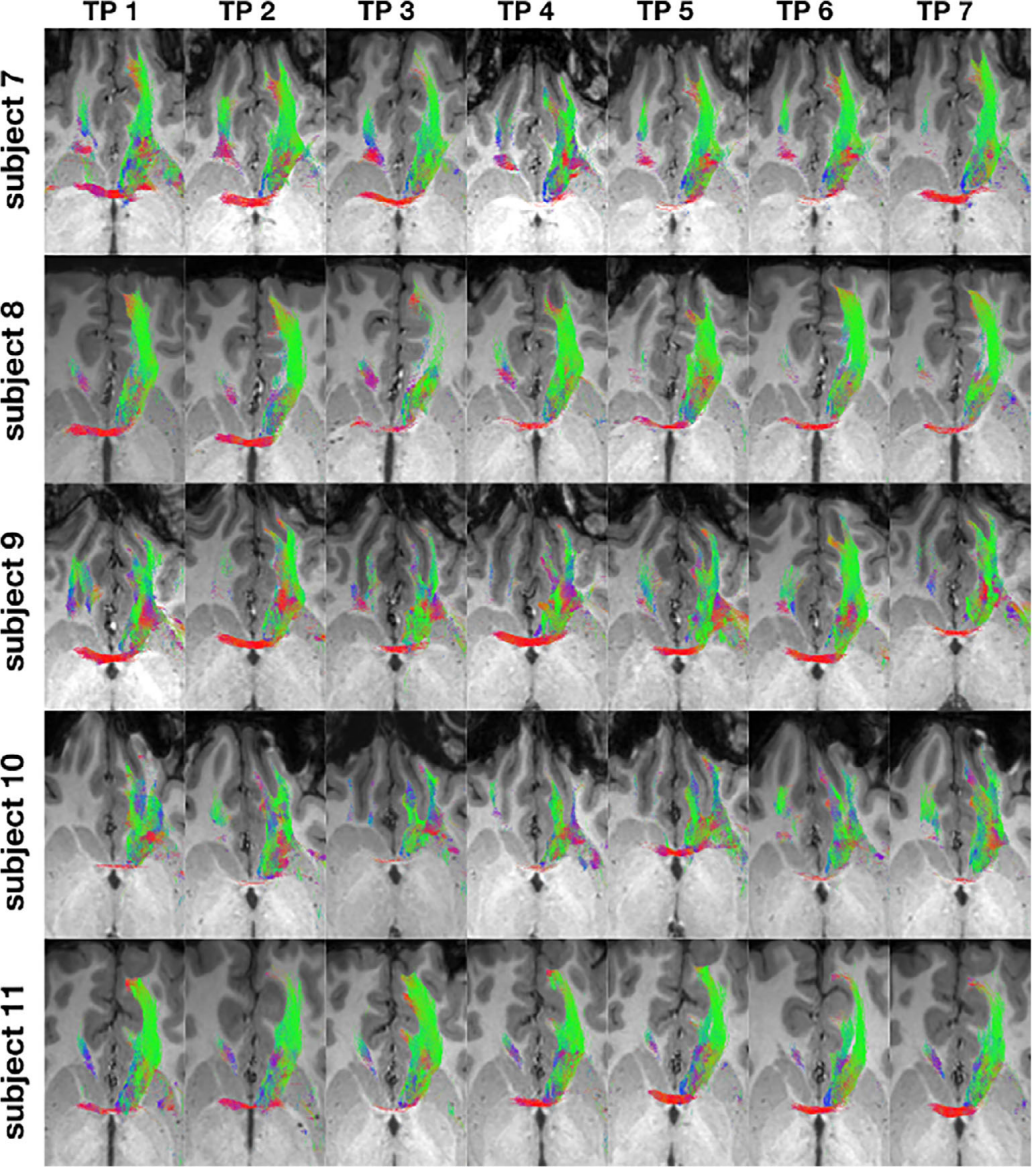
Fiber‐connections NAc–mPFC for the left hemisphere of subject 7–11 for timepoint 1–7

**FIGURE 13:**
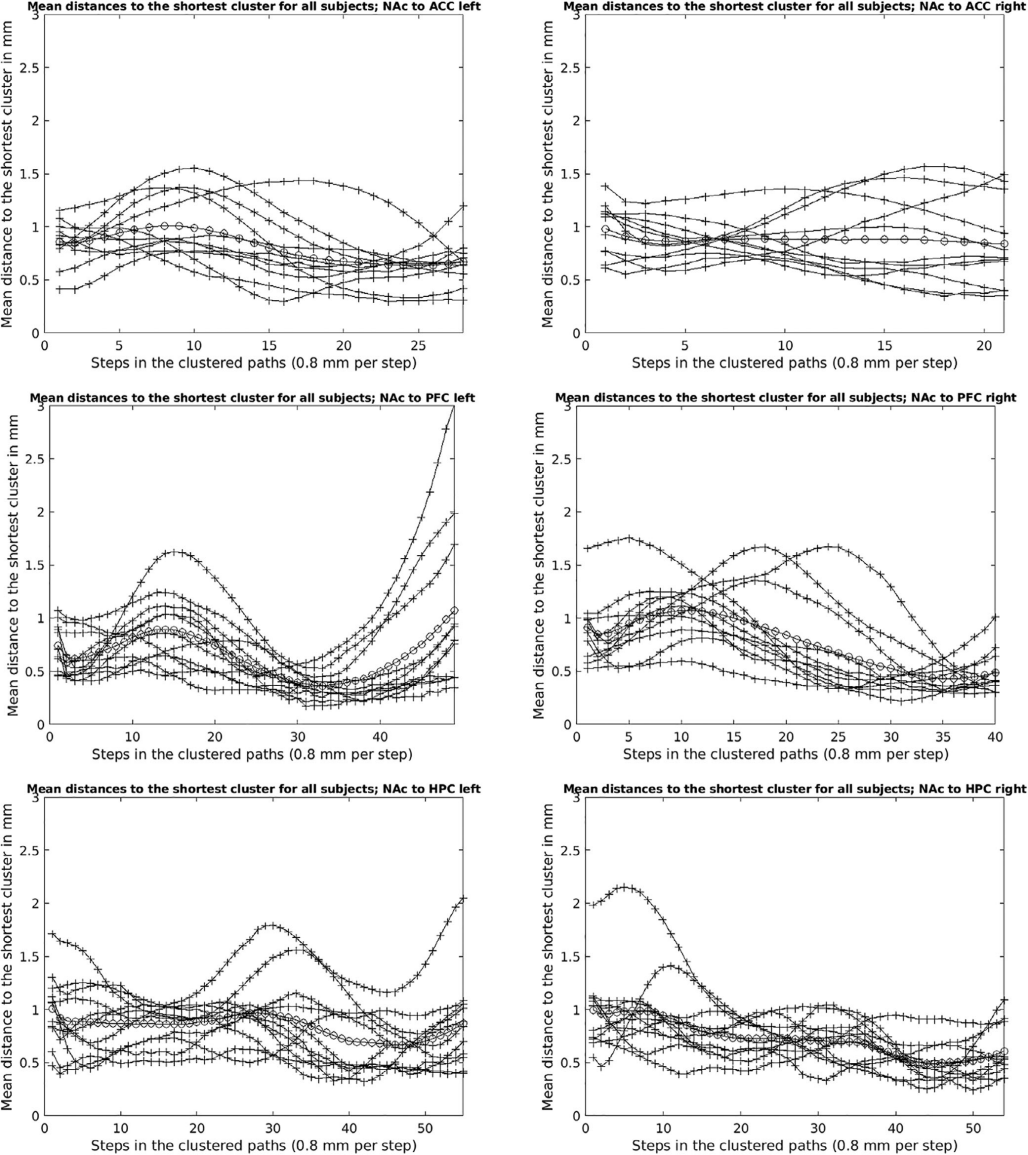
Mean distances (lines with crosses) to the shortest cluster (TP 1–7) for all subjects 1–11 and overall average distance (line with circles) across all subjects (1–11) and TP 1–7 of the NAc to ACC, PFC and HPC fiber‐connections for the left and right hemisphere in mm

**FIGURE 14:**
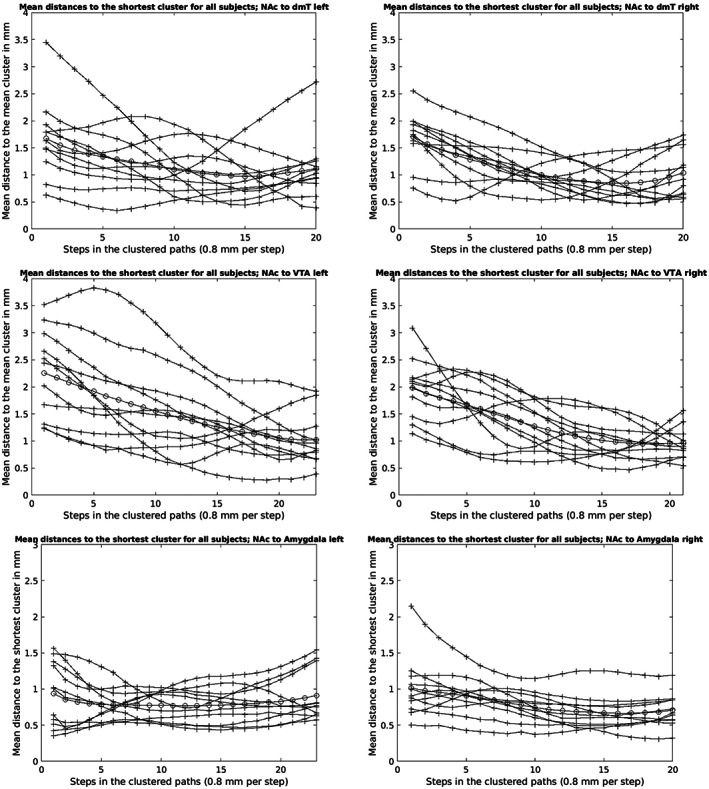
Mean distances (lines with crosses) to the shortest cluster (TP 1–7) for all subjects 1–11 and overall average distance (line with circles) across all subjects (1–11) and TP 1–7 of the NAc to dmT, VTA and AMY fiber‐connections for the left and right hemisphere in mm

**FIGURE 15:**
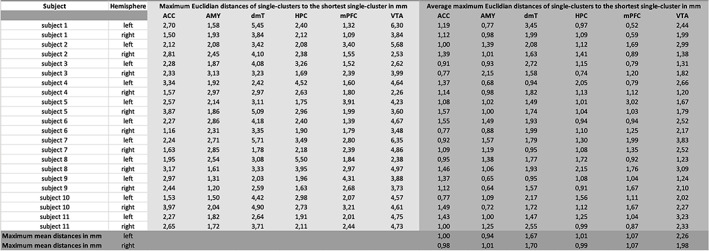
Maximum Euclidian distances of single‐clusters to the shortest single‐cluster, average maximum Euclidian distances of single‐clusters to the shortest single‐cluster and maximum mean distances in mm for all NAc to target connections of each subject 1–11 (TP 1–7) for the right and left hemisphere

- **Qualitative‐visual intra‐subject‐comparison of the NAc to mPFC fiber‐connections**
  Figures 11 and 12 illustrate the NAc to mPFC fiber‐connections of the left hemisphere for each subject 1–11 for timepoints 1–7 in axial slices at the level of the AC. This image shows a broad accordance among subjects for all fiber‐trajectories described under point 3.1.5. Minor deviations were related to the representation of a contralateral fiber‐pathway over the AC (compare as an example Figure 11 subject 1 TP 1,2,3,4,6,7 with TP 5) or were expressed by differences of the visually‐quantitatively recorded total fiber quantity within an ROI (compare fiber quantity at the level of Gyrus rectus and MOG between TP 3 and 4 of subject 5 in Figure 11).
- **Quantitative intra‐subject‐comparison of all NAc to target connections**
  Within the quantitative intra‐subject‐comparison, the following maximum Euclidian distances from single‐cluster to the shortest single‐cluster could be determined for the individual NAc‐target connections (see Figure 15): ACC 3.97 mm (subject 10, right hemisphere), AMY 3.13 mm (subject 3, right hemisphere), dmT 5.71 mm (subject 7, left hemisphere), HPC 5.5 mm (subject 8, left hemisphere), mPFC 4.31 mm (subject 9, left hemisphere) and VTA 6.35 mm (subject 7, left hemisphere). The average maximum Euclidian distances of the single‐clusters to the shortest single‐cluster for the individual NAc‐target connections were (see Figure 15): ACC 1.57 mm (subject 5, right hemisphere), AMY 2.15 mm (subject 5, right hemisphere), dmT 3.45 mm (subject 2, left hemisphere), HPC 2.15 mm (subject 8, right hemisphere), mPFC 3.02 mm (subject 5, left hemisphere) and VTA 3.83 mm (subject 7, left hemisphere). The maximum mean distances (average maximum distance of single‐clusters for all subjects 1–11 and TP 1–7 per NAc‐target‐connection) were as follows (see Figures 13, 14, 15): NAc‐ACC 1.00 mm left and 0.98 mm right hemisphere, NAc‐AMY 0.94 mm left and 1.01 mm right hemisphere, NAc‐dmT 1.67 mm left and 1.70 mm right hemisphere, NAc‐HPC 1.01 mm left and 0.99 mm right hemisphere, NAc‐mPFC 1.07 mm for both hemispheres and NAc‐VTA 2.26 mm left and 1.98 mm right hemisphere.

#### 3.3.2 Inter‐subject‐comparison using the example of the NAc to mPFC fiber‐connections

There was a large correspondence of the fiber‐connections between subject 1–11 and the respective point in time 1–7. There were inter‐individual differences in particular with regard to the representation of the contralateral fiber‐pathway via the AC (compare Figure 11 subject 5 TP 5 with subject 3 TP 3) or the visually‐quantitatively recorded total fiber quantity within an ROI (compare Figure 12 subject 10 TP 4 with subject 8 TP 4).

## 4 DISCUSSION

### 4.1 Literature review

Tract‐tracing and fiber‐dissection studies are supposed to be the “gold‐standards” for the visualization of neuronal fiber connections of the brain. Naturally, these methods can only be applied in vivo. Fiber‐tracking, the noninvasive alternative for clinical studies is error‐prone for various reasons (Alhourani & Richardson, 2015). One systematic approach for a standardized segmentation and connectivity analysis, which can minimize possible error sources is the determination of realistic functional and anatomical connections using the information from tract‐tracing and fiber‐dissection studies (Lambert et al., 2012). In preparation of the present analysis we extracted from a database containing to our knowledge the relevant literature all in vivo studies, which had reliably verified anatomical fiber connections, for example by tract‐tracing. Afferences and efferences of the NAc determined in this way were concordant with results of published reviews addressing this topic (Neto, Oliveira, Correia, & Ferreira, 2008; Park et al., 2019; Salgado & Kaplitt, 2015; Sesack & Grace, 2010; Swanson & Cwan, 1975). The majority of the studies, which we had finally selected based on brain preparations of macaques and rodents. Even though the information obtained from these publications cannot be transferred to the human brain on a one‐to‐one basis, the start and end points of the defined fiber‐connections correlated very well with the results of human‐based studies (Saleem, Price, & Hashikawa, 2007; Salgado & Kaplitt, 2015; Tsao, Moeller, & Freiwald, 2008).

### 4.2 MRI‐datasets, segmentation and fiber‐tracking

The healthy subjects included in the present study had an average age of 29 years. Because of the low mean age combined with unremarkable MRI‐images at the time of study examination, structural changes of myelinated axons were almost excluded. This ensured reliable MRI‐based imaging of NAc‐connections of the healthy brain. In patients presenting with neuro‐psychiatric or neurodegenerative diseases, however, the conditions could be different compared to the brains of healthy study participants. Structural changes associated, for instance, with brain atrophy can lead to focal changes in the fractional anisotropy and subsequent misinterpretation of diffusion‐weighted MRI‐based fiber‐connections (Lacalle‐Aurioles et al., 2016). This problem will be addressed by our group in subsequent clinical studies.

The manual segmentation of the seed‐ and target‐regions (VOIs) was carried out in the T1‐single‐subject‐template (AMY, HPC, NAc) and FLAIR‐single‐subject‐template (VTA) for each subject. The segmentation in the single‐subject‐templates instead of the T1‐ and FLAIR‐single‐subject‐datasets had several advantages: The significantly higher signal‐to‐noise‐ratio on the single‐subject‐templates compared to the single‐subject measurements improved the recognition and delineation of anatomical structures. Since the segmentation was based on landmarks, we were able to define the boundaries of the VOIs more exactly, which reduced segmentation errors. As a consequence, the seed‐ and target‐region had to be determined only once per subject and could then automatically be transferred to the other measurement‐timepoints using various calculation algorithms. In addition, the segmentation was not falsified by a potential intra‐observer variability. Potential pitfalls of manual segmentation of anatomical VOIs causing inaccuracies to a certain degree are: The total number of segmented slices, the slice‐level at the beginning or end of the segmentation, and/or the distinctness and visibility of the boundaries of a particular VOI. Instructions for MRI‐based manual segmentation were published for NAc (Lucas‐Neto et al., 2015; Neto et al., 2008), AMY (Entis, Doerga, Barrett, & Dickerson, 2012) and HPC and were widely considered for the actual analysis. To our knowledge, MRI‐based segmentation of the dmT or the VTA has not been published yet. Due to the lack of characteristic anatomical landmarks or boundaries, manual segmentation of cortex areas is comparably difficult. Thus, we opted in the case of mPFC and ACC for automatic segmentation. The utilized software, however, permitted only segmentation based on previously defined sulci and gyri. Because published information on the overall extent of the mPFC is relatively inconsistent we used for the present analysis mPFC surface patterns and landmarks elaborated by Rodrigues et al. (Rodrigues et al., 2015).

The quality of diffusion‐weighted MRI‐based tractography is theoretically determined by various ex‐ and intrinsic errors. Major extrinsic errors are, for instance, various sources of interference, which naturally occur when MR‐scans are taken from a living subject such as movement artifacts (e.g., heartbeat, breathing movements) and technical interference sources (eddy current distortions, susceptibility artifacts). Appropriate computing algorithms such as the here used “FSL‐Tool *eddy correct*” reduce these artifacts to a larger degree. Unfortunately, no data was acquired in reverse phase direction (P ➔ A) during the initial subject measurements, so that processing steps such as *FSL's topup/eddy* (Andersson, Skare, & Ashburner, 2003; Andersson & Sotiropoulos, 2016; Smith et al., 2004) could not be carried out. These are superior to the postprocessing method we used (application of field maps to reduce EPI‐ and susceptibility‐induced distorsions).

Another point is a method‐related intrinsic blur caused by an unspecified diffusion profile of the corresponding water molecules in the area of the neuronal fibers, which cannot be modified by postprocessing. Thomas et al. (2014) addressed this problem investigating the brain of macaques ex‐vivo with extremely high‐resolution diffusion‐weighted MRI scans and various algorithms for tractography. Neuronal fiber‐connections generated this way were finally compared with known axonal fiber‐projections based on tract‐tracing studies of the macaque's brain. Interestingly, independent from the applied tractography algorithm, the anatomical accuracy was insufficient. In particular, a high sensitivity of the generated fiber‐connections (high rate correct‐positive) corresponded to low specifity (high rate false‐positive) and vice versa. Hence, the authors concluded, that an anatomically valid diffusion‐weighted MRI‐based fiber‐pathway reconstruction was not sufficiently possible, especially if connections were not previously characterized. Consequently, they recommended verification and validation using corresponding postmortem tractography‐data of either tract‐tracing or fiber‐dissection studies.

Further process‐specific limitations of diffusion‐weighted MRI‐based tractography are:

1. No distinguishability between afferents and efferents of the reconstructed fiber‐tracts.
2. Incorrect calculation of the main‐diffusion vector at fiber crossing or branching points when using diffusion tensor imaging (DTI) based on a single tensor to describe multiple fibers within a single voxel (Auriat, Borich, Snow, Wadden, & Boyd, 2015; Basser, Pajevic, Pierpaoli, Duda, & Aldroubi, 2000).
3. Over‐sprouting

The diffusion of water molecules parallel to neuronal pathways is higher than perpendicular to it. For this reason, detection of fiber‐tracts by diffusion‐weighted MRI is possible at all. A measurement of the exact direction of movement of the individual water molecules (forward or backward) is not possible, only that a change in location has occurred. Therefore, a distinction between afferents and efferents of diffusion‐weighted MRI‐based fiber‐tracts is not possible, as mentioned under point 1. For a sufficient and detailed diffusion‐weighted MRI based NAc fiber‐tract reconstruction as the aim of this study, however, this is negligible. A labeling of the fiber‐tract, whether it is an afference or efference, is ultimately carried out in correlation with the reviewed basic literature, for example tract‐tracing studies.

To avoid an incorrect calculation of the main‐diffusion vector at fiber‐crossing or ‐branching points, as described in point 2, the outdated tensor model for modeling diffusion‐weighted MRI based fiber‐tracts is no longer used in the underlying study. Instead, a tensor‐free orientation‐ and distribution‐function is determined in each voxel (constrained spherical deconvolution, CSD; see also 2.3.1. [Tournier, Calamante, & Connelly, 2007]) which is much more stable and sensitive to multiple intra‐voxel fiber‐pathway trajectories (Tournier, Mori, & Leemans, 2011). The more diffusion‐directions are measured, which are distributed as evenly as possible on a spherical surface (fiber orientation distribution, FOD [Tournier et al., 2007]), the more directions per voxel can be modeled. In addition, no information is lost, as is the case with the DTI‐model, since here an average value of the individual single tensors is formed (Tournier et al., 2011). We measured 60 directions of diffusion.

Finally, if the maximum fiber‐tract length in probabilistic fiber‐tracking is chosen to high, this leads to incorrect fiber‐tracts beyond the target structure (over‐sprouting, point 3), fiber‐loops or fiber‐pathways via indirect relay‐stations and thus incorrect diffusion‐weighted MRI based fiber‐reconstructions. To minimize this problem, we tested different maximum fiber‐lengths for each of the six seed‐target‐pairs, evaluated them optically and ultimately defined the optimal fiber‐lengths for each specific fiber‐connection.

### 4.3 Anatomical validation of fiber‐connections

In general, fiber‐connections elaborated in the actual study matched closely relevant data on the course of fiber‐pathways from in‐vivo studies and from MRI‐examinations of the human brain (Mori, 2005). More in detail, the course of a significant proportion of fibers from the NAc to the dmT running together with fibers of the stria medullaris thalami (sm) or of fiber‐connections between NAc and ACC parallel to the ventral portion of the diagonal branch of Broca (VDB) has been described by Baydin et al. using fiber‐dissection technique (Baydin, Yagmurlu, Tanriover, Gungor, & Rhoton Jr., 2016). Fiber‐connection studies had also confirmed projections from the NAc to the mPFC. A more *medial* fiber bundle as identified in the present analysis, for instance, followed in rostral direction exactly the course of the fasciculus accumbofrontalis as described by Rigoard et al. (2011). Other fibers originating in the *medial* NAc with a dorsolateral course and destination in the lateral AMY projected onto a connection, which was described by Baydin et al. and was according to Rigoard et al. synonym to the Fasciculus amygdaloaccumbens (Baydin et al., 2016; Rigoard et al., 2011). Also, the course of fibers with origin in *dorsal* parts of the NAc beside the septal area projecting onto the precommissural fornix and terminating in the HPC was confirmed by dissection studies (Baydin et al., 2016). Functionally, this pathway belongs to a hippocampo‐prefrontal network, in which the NAc acts as a relay‐station between the hippocampus and the PFC. For fiber‐tracts connecting the NAc to the VTA in the present study we could not identify a morphological correlate in the current fiber‐dissection‐studies. However, MacNiven, Leong, and Knutson (2020) described two similar main fiber connections, which they refer to as inferior and superior NAc‐VTA tract in their DWI‐ and tractography‐based structural analysis of the medial forebrain bundle and results from numerous tract‐tracing‐studies also confirmed this particular pathway (Beckstead, Domesick, & Nauta, 1979; Gorbachevskaya, 1991; Herkenham, Edley, & Stuart, 1984; Zahm & Heimer, 1993). Moreover, Coenen et al. had entirely characterized the medial forebrain bundle (MFB) for the human brain using MRI and global fiber‐tracking (Coenen et al., 2018).

### 4.4 Reliability and stability of fiber‐connections

The data from the qualitative‐visual intra‐subject‐comparison of the present study revealed an overall broad concordance with the fiber‐pathway‐anatomy reported in the literature. Intra‐individual differences identified in the comparison analysis referred mainly to the total amount of fibers within specific fiber‐tract sections. Three points could be responsible for this discrepancy: (i) With measurements at different timepoints it is impossible to keep exactly constant measurement conditions referred to position‐differences, movement‐ and/or susceptibility artifacts. (ii) The calculation algorithms used for fiber‐tracking are a probabilistic method, which can cause discrete differences. (iii) The number of calculated fibers might not necessarily correlate with the actual physical characteristics of the addressed fiber‐pathway, an argument, which was raised by Nucifora, Verma, Melhem, Gur, and Gur (2005) and Vernooij et al. (2007) in context with the analysis of fiber‐pathway‐asymmetries of the Fasciculus arcuatus.

The methodology used for quantitative intra‐subject‐comparison in the present study was basically selected with respect to demonstrate stable reproducibility of the NAc fiber‐tract‐reconstructions and a prospective clinical application of the here elaborated workflow. For this purpose, the fiber‐tracts of the individual NAc‐to‐target connections were clustered for each session 1–7 of subject 1–11 and finally the Euclidian distances of the single‐clusters to the shortest single‐cluster were determined point by point. This made it possible to quantify the spatial deviations of the individual NAc‐to‐target connections between the individual sessions. The lower the distances, the better the spatial fiber‐tract match and the higher the stability of the fiber‐tracts under measurement repetition. As described in Section 2.3.2, for the sufficient clustering of some NAc‐to‐target connections (mPFC, ACC and HPC) an additional bundling/filtering of the fiber‐tracts by defining specific ROIs was necessary. Because the ROIs were placed exclusively in the course of real fiber‐tract points verified by our literature research of the basic anatomical literature and the MRI Atlas of Human White Matter (Mori, 2005), we do not consider this to represent arbitrary interference with the anatomy of the reconstructed fiber‐tracts.

The largest maximum Euclidian distance of a single‐cluster overall was 6.35 mm (see Figure 15) for the NAc‐VTA‐connection of subject 7 for the left hemisphere. A spatial fiber‐trajectory deviation in measurement repetition in this size range would be critical for sufficient and accurate fiber‐tracking based electrode positioning in DBS. However, it must be noted that the largest Euclidian distance of a single‐cluster mentioned above is a point maximum value. This means that this value reflects the maximum fiber‐pathway deviation of a single fiber‐path section of all examined NAc‐to‐target‐connections. Moreover, the Euclidian distances of the single‐clusters were relatively high especially near the seed‐ (proximal fiber‐pathway section) or target‐regions (distal fiber‐pathway section). This can be explained by the punctually different fiber trajectories exits (seed‐region) or fiber trajectories entrances (target‐region) and the resulting inhomogeneous clustering of the fibers at this location. However, since electrode placement immediately in the seed‐ or target‐region is not favored (see current study situation in the text sections below), the larger Euclidian distances here are practically less problematic for diffusion‐weighted based electrode positioning. Compared to the Euclidian distances of the single‐clusters, the average Euclidian distances averaged over all sessions 1–7 of all subjects 1‐11(maximum mean distances) for the different NAc‐to‐target‐connections were, however, distinctly lower. The maximum value corresponded to 2.26 mm for the NAc‐VTA‐connections of the left hemisphere. For some NAc‐to‐target‐connections even <1 mm (NAc‐ACC right hemisphere, NAc‐AMY left hemisphere, NAc‐HPC right hemisphere; see Figures 13, 14, 15). Overall, the individual NAc‐fiber‐tract‐connections showed high stability and reproducibility. If a maximum deviation between intended (calculated) and final target of ≤2 mm is defined as precise for frame‐based electrode positioning (Li, Zhang, Ye, & Li, 2016), then in the context of our tractography‐based workflow, the average maximum total deviation would be ≤4.26 mm (provided we define the maximum average Euclidian distance of the NAc‐VTA‐connection of the left hemisphere (see above) as the maximum deviation to be assumed) and thus similarly feasible and safe compared with other studies investigating the accuracy of electrode placement in DBS (Burchiel, McCartney, Lee, & Raslan, 2013; Ferroli et al., 2004; Fiegele et al., 2008; Lumsden et al., 2013). Moreover, the white matter tract or fiber‐tract identified as the target structure itself has a certain overall extent and therefore a volume. The fiber‐tract reconstructed via diffusion‐weighted‐MRI and subsequently clustered is line‐shaped and does not represent a volume in this sense. Even in the case of spatial deviations of the single‐clusters it can be assumed that they actually lie in the volume of the target structure. Independently, one would still perform a complementary visual inspection of the reconstructed fiber‐tracts as part of surgical planning and identify major anatomic abnormalities, if any. In addition, the diffusion‐weighted MRI‐based electrode placement approach also highlights recent research findings that clinical outcome after DBS is better when directly stimulating the functional fiber‐pathway rather than the target structure. For example, Fenoy and Schiess (2017)) described better clinical results for reducing arm tremor when directly stimulating the dentato‐rubro‐thalamic tract instead stimulating the ventrolateral thalamus itself. Moreover, Leoutsakos et al. (2018) reported a possible positive clinical effect of direct bilateral stimulation of the fornix in mild Alzheimer‐type dementia. Finally, in a noncontrolled study, Bewernick et al. (2012) showed that patients with severe refractory depression (major depressive disorder, MDD) improved significantly faster with direct electrical stimulation of the medial forebrain bundle (MFB) than with stimulation of the NAc, ventral striatum (VS)/ventral capsule (VC), or anterior limb of the internal capsule (aic). Once achieved, the positive effect on depression was stable over the long‐term. The same working group already pointed out in 2017 that possibly also in OCD‐patients (OCD = obsessive–compulsive disorder) direct MFB‐DBS could be more effective than electrical stimulation of the brain areas (NAc, VS/VC, aic) previously approved for treatment. This observation is supported by data recently published by Liebrand et al. (2019). These authors reported a correlation between the response of OCD‐symptoms and the distance of the active electrode contacts to fibers of the MFB (shorter distance = better outcome) in DBS of the ventral portion of the aic for the treatment of OCD.

In addition, as part of the quantitative intra‐subject‐comparison, we evaluated additional parameters aiming at the statistical comparability of diffusion‐weighted based fiber‐reconstructions (fractional anisotropy [FA‐maps] and density‐maps). Since the number of fibers and the fiber‐pathway within a defined VOI depend on many different framework conditions (e.g., movement and susceptibility artifacts), these parameters were not robust enough from this point of view to provide conclusive results. This experience was in line with statements formulated by Bartlett and Frost (2008), who addressed in their work the analysis of measurement errors in the context of reliability.

## 5 CONCLUSIONS

In the present study, we were able to visualize reproducible, precise and very specific projections from and to the NAc using DWI‐MRI and fiber‐tracking. A limitation of this work is the fact that the fiber reconstructions based on young and healthy subjects. Further investigations on patients will clarify if older age and associated brain atrophy or neuropsychiatric diseases reducing the volume of the NAc such in heroin addicts may influence the stability and validity of fiber reconstructions (Muller et al., 2015). The methodological workflow developed for the present study could be prospectively implemented in the clinical routine for stereotactic treatment planning and could selectively guide the implantation of DBS‐brain electrodes into the NAc. Besides local effects in the immediate vicinity to the stimulation site DBS is supposed to have preferentially a modulatory impact on diseased neuronal networks favoring myelinated axons and hence white matter as target structures (Ashkan, Rogers, Bergman, & Ughratdar, 2017; Ranck Jr., 1975).

Functional neuroimaging and connectivity analyses gave some hints that the clinical heterogeneity of OCD, for instance, is linked to distinct neural correlates (Senova et al., 2019) implying selective electrical stimulation of fiber connections depending on the individual symptom or neurocognitive pattern. Hence, diffusion‐weighted MRI‐based visualization of distinct fiber‐connections for a more personalized placement of DBS brain electrodes seems logical.

## Data Availability

Data available on request from the authors.
